# Ascending aortic aneurysm exposed to direct impingement of eccentric flow jets through a tilting-disc valve prosthesis

**DOI:** 10.1093/jscr/rjz127

**Published:** 2019-05-09

**Authors:** Hiroshi Nagamine, Yusuke Date, Takeshi Takagi, Yushi Kawase

**Affiliations:** Department of Thoracic and Cardiovascular Surgery, Yokohama Sakae Kyosai Hospital, Yokohama, Japan

## Abstract

Single-leaflet tilting-disc aortic valve prostheses are known to generate eccentric transvalvular flow jets. These prostheses are routinely inserted with the major valve opening directed toward the non-coronary sinus to achieve more favorable hemodynamic performance. From the viewpoint of blood flow dynamics, the structural and functional properties of tilting-disc aortic valves resemble those of congenital bicuspid aortic valves with right- and left-coronary leaflet fusion, which have been associated with aortopathy in the ascending aorta. Here we describe the case of a patient who had undergone aortic valve replacement in 1987 with a Björk-Shiley tilting-disc valve and required reoperation for ascending aortic aneurysm 29 years later. Eccentric flow jets through the tilting-disc valve directly impinged on the posterior wall of the ascending aorta including the aortotomy suture line, possibly contributing to the development of the saccular aneurysm in the ascending aorta.

## INTRODUCTION

Single-leaflet tilting-disc aortic valve prostheses (e.g. Björk-Shiley, Medtronic-Hall, and TTK Chitra) are known to generate eccentric transvalvular flow jets [1]. These prostheses are routinely inserted with the major valve opening directed toward the non-coronary sinus to achieve more favorable hemodynamic performance [2, 3]. From the viewpoint of blood flow dynamics, the structural and functional properties of tilting-disc aortic valves appear to resemble those of congenital bicuspid aortic valves (BAV) with right- and left-coronary leaflet fusion, which have been associated with aortopathy in the ascending aorta. Here we describe the case of a patient who had undergone aortic valve replacement (AVR) in 1987 with a Björk-Shiley tilting-disc valve and required reoperation for ascending aortic aneurysm 29 years later. Eccentric flow jets through the tilting-disc valve directly impinged on the posterior wall of the ascending aorta including the aortotomy suture line, possibly contributing to the development of the saccular aneurysm in the ascending aorta.

## CASE REPORT

**Figure 1:**
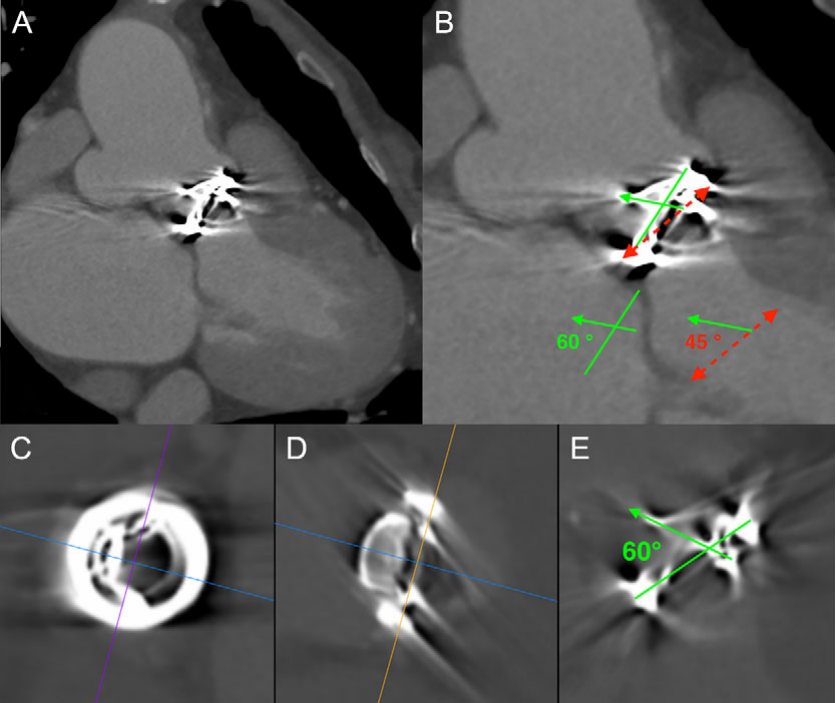
(**A**) Cardiac computed tomography (CT) angiography revealed a saccular ascending aortic aneurysm (maximum transaortic diameter: 51 mm) arising from the posterior ascending aortic wall just above the sino-tubular junction. (**B**) Cardiac CT angiography (systolic images) confirmed that the Björk-Shiley valve was tilted by the elevation of the prosthesis in the right-coronary sinus. Red dashed line: Aortic annulus (Basal ring), Green solid line: Prosthetic valve ring, Green solid arrow: Prosthetic valve leaflet. Physiologic opening angle calculated between the leaflet of the prosthetic aortic valve and the aortic annular plane was 45 degrees. (C, D, and E) Prosthetic heart valve assessment with cardiac CT (systolic images). Short axis view (**C**), frontal view (**D**), and lateral view (**E**) of the Björk-Shiley spherical-disc valve in the aortic position. Multiplanar reconstruction (MPR) image analysis revealed a normal leaflet opening angle of 60 degrees.

**Figure 2:**
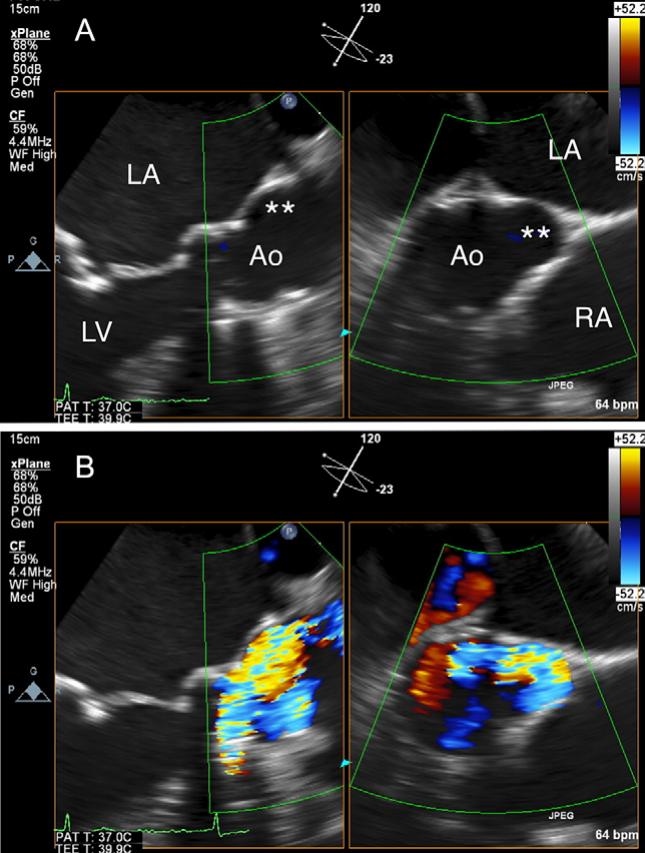
(**A**) Transesophageal echocardiography (TEE) revealed a saccular ascending aortic aneurysm arising from the posterior ascending aortic wall just above the sino-tubular junction. (**B**) TEE with color Doppler revealed eccentric systolic flow jets through the tilting disc valve prosthesis directly impinging on the saccular aneurysm. Ao: aorta; LA: left atrium; LV: left ventricle, RA: right atrium; Double asterisk: saccular aneurysm.

An 80-year-old woman, who had undergone aortic valve replacement with a 23-mm Björk-Shiley valve and open mitral commissurotomy at our hospital for multivalvular rheumatic heart disease at age 51, had chest tightness during exertion after many years of uneventful, asymptomatic clinical development. Follow-up transthoracic echocardiographic examinations over the past several years showed a normal left ventricular volume and ejection fraction, and moderate mitral stenosis (mitral valve area: 1.5 cm^2^) and an elevated peak prosthetic aortic jet velocity of 300-350 cm/s had been maintained within the boundary area without worsening. Cardiac computed tomography (CT) angiography revealed no significant obstructive coronary disease, but an unexpected saccular ascending aortic aneurysm (maximum transaortic diameter: 51 mm) arising from the posterior ascending aortic wall was observed (Fig. 1A). Transesophageal echocardiography (TEE) revealed eccentric systolic flow jets through the tilting disc valve prosthesis directly impinging on the saccular aneurysm (Fig. 2A and B, Video 1). In addition, cardiac CT angiography (systolic images) and TEE confirmed that the Björk-Shiley valve with a normal leaflet opening angle (Fig. 1C–E) was implanted with its major opening directed toward the non-coronary sinus, and was unpredictably tilted by the elevation of the prosthesis in the right-coronary sinus (Fig. 1B). The patient underwent reoperation for her ascending aortic aneurysm and possible stenosis of the prosthetic aortic valve. The ascending aorta was opened after aortic cross-clamping, and the prosthetic aortic valve and aortic aneurysm were inspected from the inside. Since the orifice of the aneurysm was located on the edge of the aortotomy suture line in the posterior wall of the ascending aorta, the lesion was probably considered a postsurgical false aneurysm. Although the Björk-Shiley valve prosthesis had no restricted leaflet motion without any obvious structural deterioration, thrombus, or abnormal pannus, it was causing an elevated transprosthetic velocity. Therefore, we decided to replace this old mechanical prosthesis with a 21-mm bovine pericardial bioprosthesis (Carpentier-Edwards Magna Ease Aortic Valve: Edwards Lifesciences, Irvine, CA, USA). The ascending aorta was replaced with a 26-mm Dacron prosthetic graft (J-Graft: Japan Lifeline Co., Ltd., Tokyo, Japan). The operative course was uneventful, and the patient recovered from surgery and has been free of complaints for almost three years.

## DISCUSSION

In the present case, preoperative TEE revealed eccentric systolic flow jets directly impinging on the saccular ascending aortic aneurysm through the tilting disc valve prosthesis. Girdauskas *et al.* demonstrated histologically that the ascending aortic wall in patients with BAV stenosis was more damaged at the point on which the systolic transvalvular flow jet impinged [4]. Barker *et al.* conducted a cardiovascular magnetic resonance imaging study on BAV syndrome and reported that the position of flow jet impingement at the ascending aortic wall corresponded to elevated wall shear stress (WSS), i.e. a well-known stimulus leading to flow-induced vascular remodeling [5]. It is therefore reasonable to assume that direct impingement of the eccentric transvalvular flow jets on the aortotomy suture line might have contributed to the development of the saccular ascending aortic aneurysm in our patient.

Björk-Shiley tilting-disc heart valves, earlier-generation mechanical prostheses widely used throughout the world in the 1970–80 s, have been withdrawn from clinical use due to structural dysfunction in some models [6]. Tilting-disc aortic valve prostheses were normally inserted with their major opening directed toward the non-coronary sinus, sutured in a supra-annular position in the area corresponding to the non-coronary sinus, and slightly tilted by the elevation of the prosthesis in the non-coronary sinus for facilitating smoother systolic transvalvular flow [2, 3]. In our patient, while the 23-mm Björk-Shiley aortic valve prosthesis with a 60-degree opening angle was actually implanted with its major opening directed toward the non-coronary sinus, it was conversely tilted by the elevation of the prosthesis in the right-coronary sinus. As a result, the functional opening angle calculated between the leaflet of the prosthetic aortic valve and the aortic annular plane was 45 degrees (Fig. 1B). Corte *et al.* quantified the restricted cusp opening of BAV (75 ± 3° for normal tricuspid aortic valves, 76 ± 3° for non-fused cusps (non-coronary cusps), and 62 ± 5° for conjoined cusps (left-coronary and right-coronary cusps)), demonstrating that the BAV’s conjoined cusp opening angle independently predicted aortic diameter and growth rate [7]. The narrow functional opening angle of the tilting disc valve prosthesis might have affected not only the elevated peak prosthetic aortic jet velocity but also the development of ascending aortic aneurysm in our case.

As for the pathogenesis of BAV-related aortopathy, there has been an ongoing nature-nurture debate between the genetic theory (i.e. the intrinsic aortic wall vulnerability causes aortopathy) and the hemodynamic theory (i.e. eccentric flow jets through a bicuspid aortic valve cause aortopathy: asymmetrical aortic dilation) [8]. If BAV-related aortopathy is caused mainly due to hemodynamic factors, asymmetrical dilatation of the ascending aorta could be assumed to develop in the same process as in late complications after AVR with a tilting-disc valve prosthesis. Re-evaluation of long-term results with tilting-disc valve prostheses in the aortic position, based on recent advances in the methodology of fluid mechanics [9, 10], is warranted in order to verify the pure hemodynamic theory of aortic aneurysm formation, excluding the influence of genetic factors.

## Supplementary Material

Supplementary Data
